# Serum Level of Cadherin-P (CDH3) Is a Novel Predictor of Cardiovascular Events Related to Atherosclerosis in a 3-Year Follow-Up Study

**DOI:** 10.3390/jcm13216293

**Published:** 2024-10-22

**Authors:** Nadezhda G. Gumanova, Dmitry K. Vasilyev, Natalya L. Bogdanova, Oxana M. Drapkina

**Affiliations:** 1 Department of Biochemistry, National Research Center for Preventive Medicine (NRCPM), 101990 Moscow, Russia; nlbogdanova@gnicpm.ru; 2 Department of Cardiovascular X-Ray Surgery, National Research Center for Preventive Medicine (NRCPM), 101990 Moscow, Russia; dvasilyev@gnicpm.ru; 3 Administrative Department, National Research Center for Preventive Medicine (NRCPM), 101990 Moscow, Russia; odrapkina@gnicpm.ru

**Keywords:** cadherin-P, CDH3, antibody microarray, serum cardiovascular biomarkers, atherosclerosis, coronary arteries, brachiocephalic–femoral atherosclerosis

## Abstract

**Background**: Placental cadherin (CDH3) is an adhesion molecule expressed in many malignant tumors. The role of serum CDH3 in atherosclerosis is unclear. **Methods**: This 3-year follow-up study measured atherosclerosis and serum CDH3 in 218 angiography inpatients. Coronary stenosis was assessed as the Gensini score. The brachiocephalic and femoral plaques were quantified by ultrasound. Microarray serum profiling was conducted in selected samples. CDH3 in the serum was measured using an indirect ELISA. The odds ratio (OR), ROC analysis, and logistic regressions were used to evaluate the associations between CDH3 content, atherosclerotic lesions, and various serum biomarkers. **Results**: Serum CDH3 was associated with the severity of atherosclerosis and diastolic blood pressure. The levels of CDH3 were able to discriminate patients with total subclinical and hemodynamically significant atherosclerotic lesions in all circulation pools (coronary, brachiocephalic, and femoral). Elevated serum CDH3 appeared to be a risk factor for cardiovascular outcomes after 3-year follow up with OR = 1.81 (95% CI: 1.07–3.72; *p* = 0.022). Endothelin-1 and NOx were associated with the content of CDH3 in the serum, suggesting the involvement of certain signal transduction pathways that may participate in plaque formation. **Conclusions**: CDH3 was associated with cardiovascular outcomes adjusted for coronary plaque presence, indicating a role of CDH3 in plaque biology.

## 1. Introduction

Cadherins are a class of transmembrane proteins important for cellular adhesion that regulate tissue organization and morphogenesis [1] and include placental cadherins (cadherin-P, CDH3), as well as multiple other cadherins. CDH3 is expressed at a high level in many malignant tumors, including colorectal cancer [2], thyroid cancer [3], and tongue squamous cell carcinoma [4]. The role of cadherin-P in cardiovascular diseases and its specific involvement in atherosclerosis are poorly understood. In a previous study, we used an antibody microarray technology to demonstrate that CDH3 is one of the biomarkers that are sex-independently associated with coronary stenosis [5]. These findings have been validated by ELISA in an external cohort [5]. The aim of the present study was to evaluate the associations of CDH3 levels in the serum of patients with atherosclerosis in the coronary, brachiocephalic, and femoral circulation.

## 2. Materials and Methods

### 2.1. Patients

The study cohort included patients (men and women aged ≥25 years) hospitalized at the National Research Center for Preventive Medicine (NRCPM) in 2018–2020. The details of hospitalization have been provided in previous publications [6,7,8]. All patients had the signs of coronary heart disease and underwent coronary angiography according to the guidelines of European Society of Cardiology [9]. Eligible patients over 25 years of age signed an informed consent for inclusion in the study and the collection and biobanking of their blood and were subjected to coronary angiography according to the indications. Indications for angiography included positive exercise test, positive stress echocardiography, symptoms of advanced angina pectoris, arrhythmia, and pathological changes in electrocardiogram with physical inability to perform exercise or stress tests, or high Duke Score.

**Figure 1:**
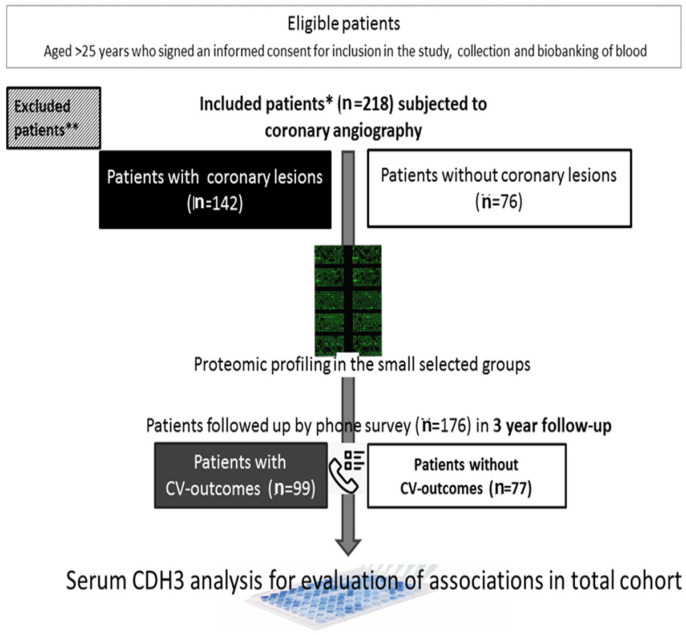
Flowchart of the study. * Included patients: Patients aged >25 years who signed an informed consent for inclusion in the study and collection and biobanking of blood and underwent coronary angiography according to indications. ** Excluded patients: Patients with myocardial infarction within 6 months of admission; any acute inflammatory disease; chronic kidney failure stage III and higher with a rate of glomerular filtration below 60 mL/min/1.73 m^2^; decompensated diabetes mellitus type I or type II with levels of glycated hemoglobin over 7.5%; left ventricular ejection fraction below 40%; any oncological disease; familial hypercholesterolemia; any hematological disease; and immune and autoimmune diseases.

After 3 years, all participants were followed up by phone survey. The survey recorded the occurrence of cardiovascular (CV) events: CV death; ischemic stroke; acute myocardial infarction; unplanned revascularization or procedure of coronary angiography at least 90 days after discharge due to the deterioration of the symptoms, including percutaneous coronary angioplasty or artery bypass grafting; and hospitalization related to deterioration due to coronary heart disease for noninvasive treatment in a cardiology department. Family history of coronary artery disease was assessed as follows: 0, no pathogenic history of CV family burden; 1, CV diseases were documented in the immediate family members (parents and siblings). Flowchart of the study is presented in Figure 1.

Exclusion criteria were as follows: myocardial infarction within 6 months of admission; any acute inflammatory disease; chronic kidney failure stage III and higher with a rate of glomerular filtration below 60 mL/min/1.73 m^2^; decompensated diabetes mellitus type I or type II with levels of glycated hemoglobin over 7.5%; left ventricular ejection fraction below 40%; any oncological disease; familial hypercholesterolemia; any hematological disease; and immune and autoimmune diseases. Additional details have been provided in our previous publications [6,7,8]. Blood pressure and heart rate were measured as described previously [6,7,8]. Angiography was performed as described previously, and the extent of atherosclerotic lesions and the calculation of the Gensini score were performed using Advantage Workstation software version 4.4 [10,11]. Peripheral atherosclerosis of the brachiocephalic and femoral arteries was diagnosed and quantified by B-mode ultrasound using a duplex sonography scanner with high-frequency (9–11 MHz) linear probes (GE Vivid 7 with TruScan raw data). The volume of the brachiocephalic and femoral plaques was assessed. A detailed description of the duplex scanning technique has been presented elsewhere [8]. Lesions of the brachiocephalic arteries were assessed as follows: intact brachiocephalic arteries (no lesions), mild stenosis with the degree of stenosis < 60%, and severe or hemodynamically significant stenosis ≥ 60% according to the guidelines of the European Society of Cardiology [12]. Lesions of the femoral arteries were assessed as follows: intact femoral arteries (no lesions), mild stenosis with a degree of stenosis < 70%, and severe stenosis ≥ 70% [13]. Smoking status was assessed as follows: 0, never smoked; 1, smoking in the past; and 2, present smoker. Statin treatment was recorded both before and after hospital admission. All patients were on the same diet pattern in a hospital setting.

Written informed consent had been signed by all patients who participated in the study. The Independent Ethics Committee of NRCPM approved the protocol of the study according to Helsinki Declaration (approval no. 09-05/19).

### 2.2. Blood Sampling

Blood was withdrawn from the cubital vein at the baseline of the study. The serum and citrate plasma were separated by centrifugation (1000× *g*, 15 min, 4 °C). The samples were processed as described previously [5].

### 2.3. Routine Blood Analysis

Routine blood tests were conducted in NRCPM according to the guidelines approved by the Center for External Quality Control of Clinical Laboratory Testing of Russian Federation (www.fsvok.ru, accessed on 21 September 2020). The analyses of total cholesterol, low-density lipoprotein (LDL)-cholesterol, high-density lipoprotein (HDL)-cholesterol, fibrinogen, C-reactive protein (CRP), insulin, glucose, leptin, endothelin, and adiponectin have been described previously [10,14] and were performed using commercial kits from Diagnostics Biochem Canada, Inc., London, ON, Canada and Invitrogen, Thermofisher Scientific, Wien, Austria.

Levels of serum NOx (nitrates and nitrites) were analyzed under a diet in a hospital setting using Griess reaction after the reduction of nitrate with vanadium (III) chloride [15] as described previously [16,17]. Reagents for the assays were from Sigma-Aldrich (St. Louis, MO, USA).

### 2.4. Microarray Analysis in the Serum

Microarray analysis of serum proteome was performed in four serum samples of group A (severe coronary stenosis with a high Gensini score) and of group B (no coronary stenosis according to the Gensini score), using antibody microarrays with 656 antibodies per slide in two replicates (ASB 600, Full Moon BioSystems, USA) as described previously [5]. All eight serum samples were selected from the study cohort.

### 2.5. Indirect ELISA of CDH3

The samples were adjusted to the same protein concentration based on the protein assay performed, as described in our previous publication [5]. The values of possible nonspecific cross reactivity were estimated by two types of control samples. Control 1 (background absorbance) comprised the samples containing immobilized serum (10 µg/mL) without the addition of primary antibodies. Control 2 (maximum binding) comprised the samples of immobilized recombinant human P-cadherin (40 pg/mL) in 100 µL of coating buffer. The optical density (OD) values were recalculated for each assayed batch according to the following equation: OD corrected = (OD450 of control 2 − OD450 of control 1)/OD450 of serum sample. The corrections and calibration curves were calculated by Magellan^TM^ software (Tecan, Switzerland).

### 2.6. Statistical Analysis

Statistica software version 8.0 and SPSS IBM statistics version 23 were used for statistical analyses. Sample size and power were estimated using the online calculator Sampsize https://sampsize.sourceforge.net/iface/s2.html#nm (accessed on 10 September 2021) for the estimation of sufficient numbers of outcomes for analysis. The Kolmogorov–Smirnov criterion was used for test of normality of the distributions. The data are shown as the mean (SD) (standard deviation). Two-tailed non-parametric analysis of variance and Kruskal–Wallis and Mann–Whitney tests were used to compare the groups. The odds ratio (OR) and area under curve (AUC) with 95% confidence interval (CI) were calculated as appropriate. Multivariate logistic regression was performed with Wald test. The chi-squared statistic corresponded to the difference in −2 log-likelihoods between the final model and a reduced model. The reduced model was formed by omitting an effect from the final model. The null hypothesis was that all parameters of that effect are zero. *p* values < 0.05 were considered significant.

## 3. Results

The study recruited a cohort of 218 patients aged 63 ± 10.9 years (54% men) who never smoked (*n* = 98; 45%), smoked in the past (*n* = 28; 12.8%), or were smokers at the time of the study (*n* = 92; 42.2%) with a Gensini score 57.0 ± 38.4 (mean ± SD). The Gensini score varied from 0 to 197 points. A Gensini score of zero (no coronary lesions) was observed in 76 (34.9%) patients; coronary stenosis was less than 50% in 71 (32.6%) patients; and multiple vessel lesions, including left main disease with stenosis over 50% of at least one coronary artery, were detected in 71 (32.6%) patients. Thus, coronary artery disease was demonstrated in 65.2% of patients. A total of 58% of patients were treated with statins before blood withdrawal.

A total of 71 (32.6%) patients out of all 218 patients subjected to coronary angiography received planned revascularization at the baseline. Only 5.2% (*n* = 4) of patients with a Gensini score of zero (total *n* = 76) received planned revascularization. A total of 71 patients (50%) with a Gensini score more than 1 (total *n* = 142) received planned revascularization. Overall, 97.3% of these patients were classified into the group of patients with severe coronary lesions with Gensini score more than 10.

Angiographic characteristics of the total cohort were as follows for all circulation pools: no lesions (*n* = 42; 15%), mild lesions (*n* = 95; 34%), and severe hemodynamically significant lesions (*n* = 144; 51%); for coronary circulation, no coronary lesions (Gensini score = 0; *n* = 76; 35%), Gensini score > 0 with mild lesions (0 > Gensini score > 36, median Gensini score; *n* = 63; 29%), and severe lesions (≥36 median Gensini score; *n* = 79; 36%); for brachiocephalic circulation, no lesions (*n* = 41; 19%), mild lesions (<60%; *n*= 156; 72%), and severe lesions (≥60%; *n* = 21; 10%); and for femoral circulation, no lesions (*n* = 69; 32%), mild lesions (<70%; *n* = 66; 30%), and severe lesions (≥70%; *n* = 83; 38%).

A total of 85% of patients of the cohort had a single or multiple plaques in any (coronary, brachiocephalic, or femoral) circulation pool, 82% of patients had a single or multiple plaques in the brachiocephalic vessels, and 68% of patients had a single or multiple plaques in the femoral vessels. Notably, a high correlation between the femoral and coronary lesions (quantified as the corresponding Gensini score) was detected (r = 0.8, *p* < 0.05), similar to the data reported previously [18]. A correlation between the brachiocephalic and coronary lesions was also significant; however, the correlation coefficients were lower (r = 0.5, *p* < 0.05).

A total of 176 (80.8%) out of 218 included participants were followed up by phone survey after 3-year follow-up, and 42 participants (19.3%) were unavailable for follow up. The survey included 99 (56%) cardiovascular events: 4 cardiovascular deaths; 4 incidents of ischemic stroke; 1 acute myocardial infarction; 45 unplanned revascularizations of coronary and peripheral arteries, including 8 coronary artery bypass grafting, percutaneous coronary angioplasty, and coronary angiography at least thee months after discharge from hospitalization due to deterioration of the symptoms; and 37 hospitalizations related to deterioration of coronary artery disease for noninvasive treatment in a cardiology department. A total of 95 cardiovascular events (excluding cardiovascular death) were united as cardiovascular outcomes for further analysis. A cardiovascular death was not included because of a low number of the incidents. Thus, only alive participants with the cardiovascular outcomes were used for calculations. General characteristics of the groups with and without cardiovascular events are presented in Table 1. Patients who underwent any type of repeated revascularization, including coronary artery bypass grafting, percutaneous coronary angioplasty, and coronary and peripheral arteries during 3-year follow up had multiple vascular lesions in multiple vessels, with a very high average Gensini score of 59.3 (upper quartile).

**Figure 2:**
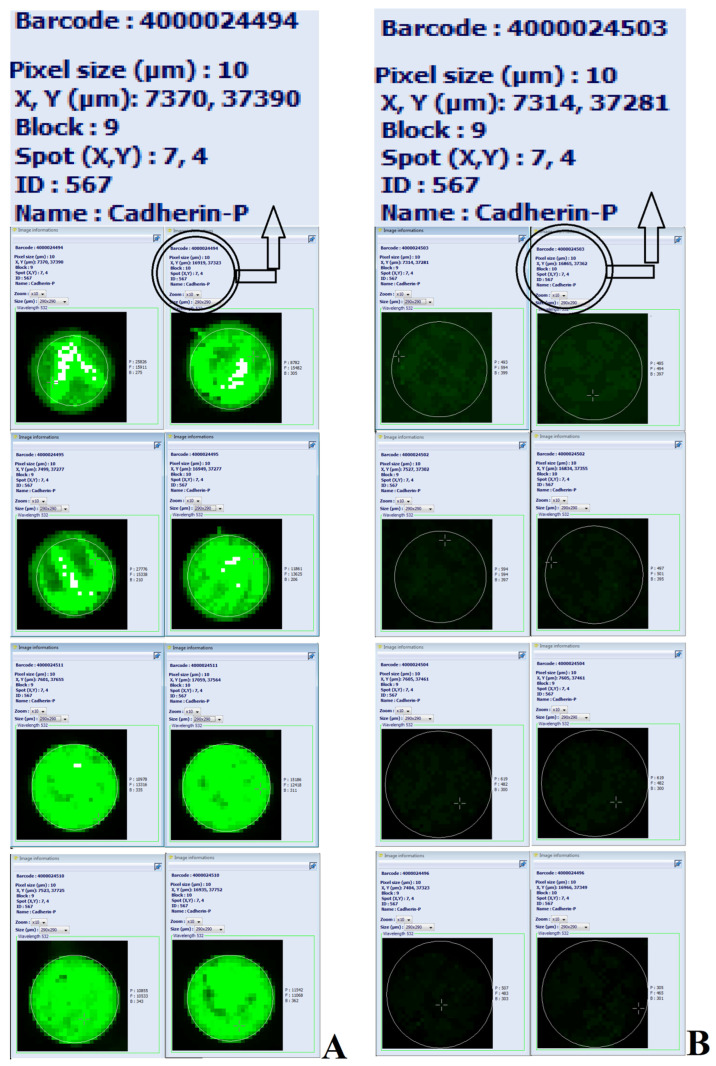
Image of eight Explorer antibody microarrays (ASB600, Full Moon Biosystems, Sunnyvale, CA, USA) with 656 antibodies per slide in two replicates for each antibody. The load of labeled serum proteins was 500 ng; scan settings: velocity: 20 l/s; laser power: 5.0; detector gain: 100; pixel size: 10; wavelength: 532 nm. The images illustrate the differences in the levels of cadherin-P in the serum of four patients with coronary stenosis in two replicates ((**A**) on the left) versus the serum of four patients with no coronary lesions in two replicates ((**B**) on the right). The text labels are enlarged and presented at the top.

Serum proteome profiling was performed in small subgroups A with coronary stenosis and B with coronary vessels without lesions validated by coronary angiography. These groups were selected from the total cohort. The levels of cadherin-P (CADH3; UniProtKB P22223) were higher in the subgroup A group compared with that in the subgroup B (Figure 2).

**Figure 3:**
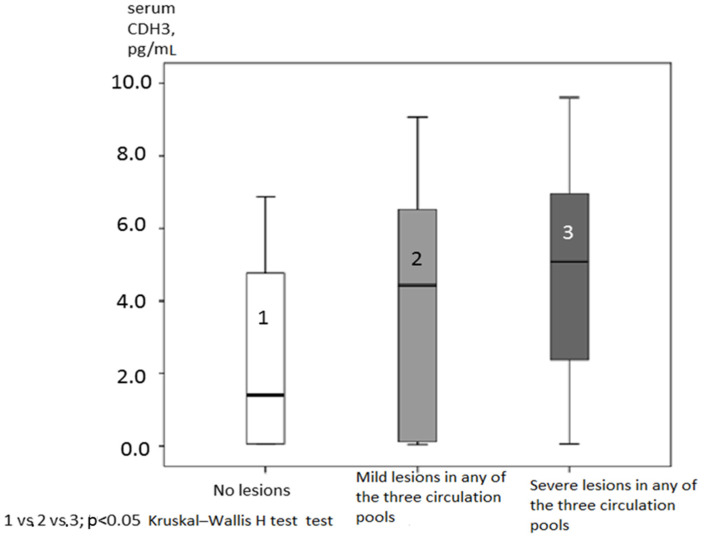
CDH3 content (pg/mL) in patients without lesions, with mild lesions, and with hemodynamically significant lesions in any of the tree circulation pools (coronary, brachycephalic, and femoral).

High serum levels of CHD3 in the total cohort were associated with mild and severe hemodynamically significant atherosclerotic lesions in any of the three types of circulation: coronary, brachiocephalic, and femoral (Figure 3). CHD3 content in patients without lesions (1), with mild lesions (2), and with severe (3) lesions in any of these types of circulation was gradually higher—2.47 ± 2.39; 3.93 ± 2.96; and 4.69 ± 2.69 pg/mL, respectively (Figure 3)—and the differences between these groups were significant (*p*_1_ = 0.009; *p*_2_ = 0.0003; and *p*_3_ = 0.045 according to Kruskal–Wallis test, respectively).

CHD3 content was associated with coronary stenosis with Gensini score 0/1 as a binary variable defined by ROC analysis: AUC 0.69; 95% CI (0.64–0.75); *p* = 0.0001. Optimal cut-off for CDH3 was 0.52 pg/mL according to the AUC values.

CHD3 content was also associated with brachiocephalic lesions at the cut-off 0 (no lesions)/1 (atherosclerotic lesions) (AUC = 0.58; 95% CI (0.51–0.64); *p* = 0.047). In the case of atherosclerotic lesions, an optimal cut-off for CDH3 was 3.9 pg/mL. CDH3 content in the groups divided according to the status of brachiocephalic atherosclerotic lesions was as follows: patients with no brachiocephalic lesions had CDH3 levels of 3.43 ± 2.6 pg/mL, and patients with any brachiocephalic lesions had CDH3 levels of 4.15 ± 2.9 pg/mL (*p*= 0.046; Mann–Whitney test).

No associations were observed between serum CDH3 and atherosclerotic lesions in the femoral circulation if other types of circulation were not considered. Thus, the levels of serum CDH3 in patients who had coronary (Table 1) and brachiocephalic atherosclerotic lesions were significantly different from those in patients with no coronary lesions and no brachiocephalic lesions, respectively.

The cohort was divided based on Gensini score and CV events, and CDH3 levels are shown in Table 1. No associations between CDH3 levels and statin treatment before blood withdrawal were observed in the total cohort or in patients who responded to the survey calls.

The association between CDH3 and CV outcomes was evaluated by comparing the serum concentrations of CDH3, which were different in patients with CV outcomes versus patients with no outcomes (Table 1). CDH3 optimal cut-off concentration (4.6 pg/mL) for the 3-year follow up of the outcomes was estimated using ROC analysis (Table 2). The CDH3 cut-off point of 4.6 pg/mL corresponds to the optimal accuracy of the ROC curve verified using OR evaluation as described in Table 2 where associations between the levels of CDH3 and combined CV outcomes were defined. Serum levels of CDH3 > 4.6 pg/mL were associated with a higher number of the incidents of CV outcomes (OR = 1.81; 95% CI: 1.07–3.72; *p* = 0.0022) (Table 2).

Binomial regression analysis was performed after a 3-year follow up period to determine whether CDH3 concentrations were independently associated with CV outcomes. Binomial logistic regression adjusted for conventional CV risk factors (age, sex, smoking, and blood pressure) and coronary lesions (Gensini score as a continuous variable) demonstrated that serum CDH3 was independently associated with combined CV outcomes (*p* = 0.013) (Table 3).

Classification tables were generated as a variant of binomial regression to evaluate whether the inclusion of CDH3 in the list of parameters improves the prediction of CV outcomes over the standard or base model with routine cardiac rick factors (sex, age, smoking, systolic blood pressure, diastolic blood pressure, and total cholesterol levels). These risk factors are the components of SCORE (Systematic Coronary Risk Evaluation) index, which is used as a predictor for CV death in 10-year follow-up. Classification tables (Table 4) were generated using dichotomous value fitting, including aggregated routine cardiac risk factors (sex, age at a median cut-off of 65 years, smoking, family history of CVD, systolic blood pressure at a median cut-off of 130 mm Hg, diastolic blood pressure at a median cut-off of 70 mm Hg, and total cholesterol at a median cut-off of 4.2 mmol/L). The upper part of Table 4 corresponds to the base model, and the lower part of Table 4 corresponds to the base model plus CDH3 (a cut-off of 4.5 pg/mL). Net reclassification improvement (NRI) was calculated using the data of Table 4 and was approximately 2%. OR for the base model plus CDH3 was calculated using the data of Table 4 (OR = 3.23; *p* = 0.0006) (Table 5). In contrast, OR for the base model without CDH3 was relatively high but was characterized by somewhat lower statistical significance (OR = 2.26; *p* = 0.017) (Table 5).

Associations of various biomarkers in the cohort, including lipid profile parameters (total cholesterol, triglycerides, HDL-cholesterol, and LDL-cholesterol), energy metabolism characteristics (insulin, HOMA-IR, glucose, leptin, and adiponectin), inflammation (fibrinogen and C-reactive protein), and endothelial functional markers (endothelin-1 and NOx) with CDH3 were analyzed.

Serum content of CHD3 was associated with NOx and endothelin-1 (Table 6). However, an association of NOx with CDH3 was not independent according to the data of multivariate logistic regression (Table 7). The level of NOx was measured in a hospital setting under a diet. Therefore, NOx can be considered a biomarker of endothelial function [19].

Notably, serum CDH3 was associated with diastolic blood pressure (Table 6), which had an independent reverse association with endothelin-1 (*p* = 0.034) in a linear regression (Table 8). Endothelin-1 is a well-known regulator of blood pressure. Notably, in contrast to CDH3 and endothelin-1, NOx was associated with systolic blood pressure (*p* = 0.045).

Other assessed biomarkers (total cholesterol, triglycerides, LDL-cholesterol, HDL-cholesterol, glucose, insulin, HOMA-IR, C-reactive protein, fibrinogen, adiponectin, and leptin) were not associated with serum CDH3 (Table 6).

Thus, our results indicated that high concentrations of CDH3 were associated with an increase in the incidents of cardiovascular outcomes, diastolic blood pressure, and the severity of coronary lesions. Elevated serum CDH3 was the predictor of cardiovascular outcomes adjusted for conventional CV risk factors and the presence of coronary plaques. Elevated serum CDH3 was associated with lesions predominantly in the coronary and brachycephalic circulation. Endothelin-1 and NOx were associated with high levels of CDH3. These biomarkers point out toward possible signal transduction pathways that regulate cellular processes participating in the formation of atherosclerotic plaques.

## 4. Discussion

Endothelial tissues are composed of the layers of cells that are connected by cell-to-cell junctions, in particular adherens and tight junctions. Cadherins are integral membrane proteins with adhesion receptor functions. Cadherins are Ca^2+^-dependent adhesion molecules that are normally engaged in homo- or heterotypic cell-to-cell interactions [20] and homeostatic functions in normal tissues [21]. Cadherin-P has approximately 67% homology with cadherin-E, with differences mainly in the extracellular region of the protein molecule [22,23]. Cadherin-P is expressed in several adult tissues and is usually coexpressed with cadherin-E. These tissues include the basal layer of the epidermis, breast, prostate, mesothelium, ovary, cervix, hair follicles, and corneal endothelium [24,25]. In addition to a provision of tissue architecture [21], cadherin-P is involved in various disease states, including specific hereditary genetic disorders and cancer [21].

The impaired expression of cadherin-P contributes to the invasive phenotype of ovarian cancer. These effects of cadherin-P are mediated by the activation of low molecular weight GTP-binding proteins, including Rho GTPases, Rac1, and Cdc42 and by an increase in p120 catenin in the cytoplasm [26,27]. Cadherin-P induces the ligand-independent activation of insulin-like growth factor-1 receptor (IGF-1R) [28]. Cadherins induce phosphorylation signaling due to their associations with other signaling systems, including receptor tyrosine kinases [29].

Cadherin-P overexpression and the poor survival of patients are known to be associated with the oncogenic signaling of the nitric oxide pathway in breast tumor. This mechanism of induction provides the activation of the Ras/MEK/ERK signaling pathway by NO, leading to the activation of Ets-1, which is a transcription factor involved in metastasis, tumor growth, and cadherin upregulation. RNA knock-down of Ets-1 suppresses the expression of cadherin-P induced by NO [21,30]. Treatment with NO decreases the level of cadherin-E and cell adhesion, indicating that NO signaling is involved in the epithelial-to-mesenchymal transition (EMT) in breast cancer [21,31], and EMT is considered the main driving force of the metastatic cascade.

The data of the present study indicated that high CDH3 was associated with cardiovascular outcomes adjusted for the presence of plaques in the coronary circulation, suggesting a role of CDH3 in plaque biology. Additionally, we demonstrated significant associations between CDH3 and the severity of coronary stenosis.

Revascularization is defined as a major factor that determines cardiovascular outcomes. It is important to discriminate between the role of CDH3 as a predictor of severe artery disease, thus being a predictor of revascularization and CV outcomes, and the role of CDH3 as an independent predictor of CV outcomes. This discrimination may be possible because our analysis used only unplanned coronary angiography at least 3 months after discharge from hospitalization, while the downstream procedures based on the presence of significant coronary stenosis were not considered in the present study.

Moreover, binomial regression analysis was performed to discriminate between the predicting roles of CDH3 in terms of the severity of coronary stenosis and CV outcomes. CDH3 had an independent impact on CV outcomes adjusted by the plaque lesions (assessed as Gensini score) in coronary circulation (Table 3), clearly indicating that CDH3 was an independent predictor of CV outcomes. Calculations of NRI for the base model of CV outcomes included only routine cardiac risk factors and CV family history. However, the addition of CDH3 to this base model resulted in NRI > 0, gaining 2% over the base model without CDH3. This result indicated that CDH3 optimized the prediction of CV outcomes by the base model of conventional cardiac risk factors. NRI is widely used to assess relative ability of two risk models to distinguish between low- and high-risk individuals. However, the validity and usefulness of NRI have been questioned. The main critical points emphasize that NRI has substantial variability that heavily depends on the risk cutoff values, which makes it unstable for the comparison of miscalibrated models. Moreover, the results of NRI may be challenging to interpret correctly. Additional criticism includes a possibility of falsely useful noninformative models and problematic evaluation of confidence intervals [32]. Moreover, a meaningful range of improvement for NRI has not been established [33], which is one of the main limitations of NRI. NRI depends on the selection and number of categories [33].

Elevated CDH3 levels were predictors of cardiovascular outcomes independently of the severity of coronary atherosclerotic lesions. Considering that 35% of patients had completely clear coronary arteries (Gensini score = 0), our results further demonstrated that these patients remain at high risk for future cardiovascular events and require optimal risk stratification and appropriate secondary prevention therapies [34,35]. Notably, in the present study, 58% of patients of the cohort were treated with statins before blood withdrawal. Thus, associations between statin therapy and CDH3 levels were examined. These parameters were not associated in the total cohort or in patients who responded to the phone survey. Thus, statin therapy is highly unlikely to have influenced the results of the present study.

Similar to other members of the cadherin family, cadherin-P regulates embryonic development, adult tissue architecture, differentiation, cellular shape and polarity, growth, and the migration of the cells [36,37,38]. The binding of various adhesion molecules to activated endothelium influences the interactions with leukocytes and monocytes important for atherosclerosis [38]. For example, the increased secretion of cadherin VE (vascular endothelial cadherin-5) from epicardial arteries is associated with the severity of coronary atherosclerosis [39,40]. Changes in the levels of cadherin VE in vivo may influence vascular permeability, induce cell growth, and promote vascular fragility [41].

**Figure 4:**
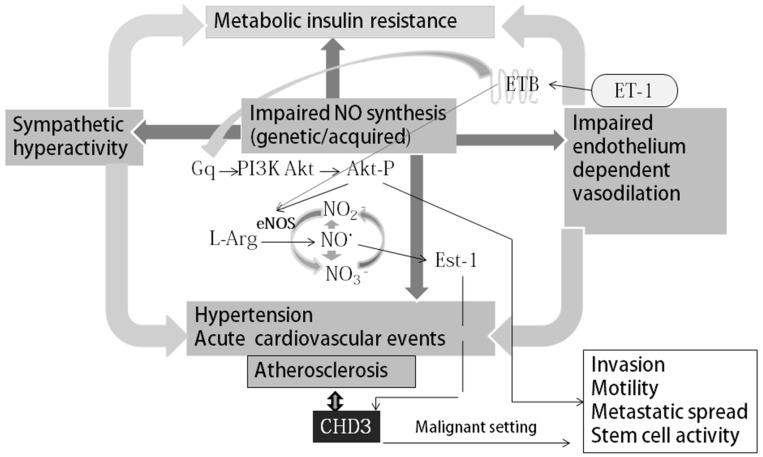
Scheme of the interactions of NO synthesis and endothelin-1 with CDH3 in the context of vascular and sympathetic abnormalities in cardiovascular diseases. Tissue hypoxia increases the production of endothelin-1, leading to an increase in the tissue and plasma levels of endothelin-1. The activation of the endothelin receptor B (ETB) leads to the activation of the pathway, resulting in the release of NO that causes vascular smooth muscle relaxation and the activation of the L-arginine/NO pathway. The overexpression of cadherin-P is linked to the oncogenic signaling of NO in human breast tumors. This mechanism involves the activation of the Ras/MEK/ERK signaling pathway by NO, in turn leading to the activation of Ets-1, which is a transcription factor implicated in metastasis and tumor progression and causes cadherin-P upregulation. The cadherin-P signaling pathway in malignant tumors influences the invasion, motility, stemness, and metastastic potential of various tissues. ET-1, endothelin-1; ETB, endothelin-1 receptor type B; PI3K, phosphatidylinositol-3-kinase; Gq, GTP-binding protein Gq; NO, nitric oxide.

The data of the present study indicated that endothelial biomarkers (endothelin-1 and NOx) were associated with serum content of CDH3, and CDH3 was associated with the degree of atherosclerotic lesions (Figure 3). These biomarkers are involved in generally interconnected signaling pathways (Figure 4). Although CDH3 was associated with the presence of atherosclerotic plaques, we have no evidence of the associations of CDH3 with plaque stability or anatomic characteristics because these data were not collected in the present study.

Cadherins are known to have important mechanical functions, which are required during the development and maintenance of epithelial or endothelial barriers [41]. For example, endothelin-1 is released in response to a number of stimuli, including acute and chronic stress, hyperosmolality, high sodium intake [42], and hypoxia [43]. Tissue hypoxia increases the production of endothelin-1 by endothelial cells or adipocytes, leading to an increase in the levels of endothelin-1 [44]. The activation of the endothelin B receptor (ETB) has been extensively investigated in vascular endothelium [45,46,47]. ETB activation results in the release of prostaglandins and NO, inducing vascular smooth muscle relaxation [48]. Moreover, plasma endothelin-1 level is positively correlated with insulin resistance in humans [47,48]. The effects of this increase in the levels of endothelin-1 in combination with elevated insulin resistance and decreased glucose uptake depend on the target tissue because the expression and activation of endothelin receptor subtypes are tissue-specific. Additional effects of insulin on the cardiovascular system are mediated by the sympathetic nervous system and the L-arginine/NO signaling pathway [49,50]. Thus, a defect of NO synthesis may facilitate sympathetic activation because NO is known to inhibit central neural vasoconstrictor outflow in animals and humans [48,49]. Additional metabolic effects of insulin target the endothelium [51,52,53]. Insulin upregulates endothelial NO synthesis and thus enhances blood flow [53]. Hence, a decrease in NO levels blocks this effect of insulin on local circulation and on glucose uptake [53]. This effect is mediated by the activation of the phosphatidylinositol-3-kinase signaling pathway and changes in the phosphorylation of endothelial NO synthase.

Overall, the present study demonstrated that NOx and endothelin-1 were associated with the serum CDH3 and systolic and diastolic blood pressure, respectively (Table 7). We suggest that NOx and endothelin-1 may act as the regulators of the serum content of CDH3, and this hypothesis is in agreement with known effects of these mediators on vascular tone [54]. The findings of the present study are supported by a scheme that illustrates the relevant signaling pathways in atherogenesis (Figure 4).

## 5. Conclusions

Elevated serum CDH3 was the predictor of cardiovascular outcomes adjusted for age, sex, coronary atherosclerotic lesions, family history of CVD, and conventional CV risk factors and was associated with atherosclerosis predominantly in the coronary circulation. Endothelin-1 and NOx were associated with the content of CHD3 in the serum, suggesting that several signal transduction pathways participate in the formation of atherosclerotic plaque. Thus, CDH3 is associated with cardiovascular outcomes adjusted for the presence of coronary plaques, indicating a role of CDH3 in plaque biology. CDH3 may be a promising marker for the assessment of cardiovascular risk. Our results demonstrated that these patients remain at a high risk for future cardiovascular events and require optimal risk stratification and appropriate secondary prevention therapies.

## 6. Limitations

The limitations of this study referred to a low number of CV death incidents, and thus, conclusions referring to the risk of cardiovascular mortality were omitted. Missing data about patients who did not respond to the follow-up could have caused a possible bias in our conclusions. Since some of the participants were unable to accurately recall the dates of the events, Cox regression analysis was not performed.

Although sample size and power were estimated using the online calculator Sampsize, a possible bias is anticipated because of reduced sample size due to the division of patients into subgroups for evaluation of the associations with Gensini score and CV outcomes. Therefore, binomial logistic regression for associations between cardiovascular outcomes and serum CDH3 was analyzed in the total cohort.

This analysis does not sufficiently address potential confounders, such as medication use, lifestyle factors, or comorbidities that could influence CDH3 levels and cardiovascular outcomes. Only statin use, smoking status, waist circumference, and BMI as lifestyle factors were included in the present analysis. Waist circumference and BMI are likely considered an indirect sign of physical activity. Metabolic syndrome was unlikely a cause of these differences because a glycated hemoglobin level over 7.5% was an excision criterion of the present study. An expanded analysis of associations between medication treatment, details of lifestyle, and CHD3 is going to be an aim of our further research. Most of potentially confounding comorbidities were excluded from the present study (see the exclusion criteria). This exclusion was intentionally aimed to clearly estimate the relationships between atherosclerosis and CDH3.

In the present study, we have used recombinant CDH3 as a standard. CDH3 is a transmembrane glycoprotein complex with various antigenic epitopes. Thus, some forms of CDH3 shedded in the blood may have unknown antigenic and structural properties due to various sources of origin. Thus, antibody reactivity of the recombinant CDH3 as a standard may differ from the reactivity of serum CDH3, suggesting a potential bias in the calibration curve. Thus, serum concentrations of CDH3 assessed using this curve may not accurately represent the actual levels of CDH3 in the serum and are provided only as an estimate.

## Figures and Tables

**Table 1:** General characteristics of the participants (N = 218) and the levels of biochemical markers in the total cohort, in the groups with or without cardiovascular (CV) events * within a 3-year follow-up, and in the groups with (Gensini score > 0) and without (Gensini score = 0) according to the data of coronary angiography. The groups were compared by Mann–Whitney U test.

	Stratification After 3-Year Follow Up			Baseline Characteristics			
Parameter	Patients with No CV Events *	Patients with CV Events		Total Cohort	Patients with Gensini Score = 0	Patients with Gensini Score > 0	
N	77	99		218	76	142	
	Mean (SD)		*p*	Mean (SD)			*p*
General characteristic							
Sex	1.48	1.46	NS	1.39	1.57	1.42 (0.49)	0.036
Age	59.7 (12.1)	63.7 (9.3)	NS	63.2 (10.9)	60.5 (11.9)	63.5 (10.1)	NS
Smoking (0/1/2) **, %	53/14/33	41/13/47	NS	45/13/42	50/14/36	42/9/46	NS
Patients with CV family history ***, %	59.7	75	0.01	67.8	47.4	81	>0.0001
BMI, kg/m^2^	28.8 (4.7)	30.1 (4.8)	NS	29.9 (5.8)	29.4 (5.6)	30.1 (5.1)	NS
WS, cm	90.4 (9.9)	94.9 (13.2)	NS	93.7 (12.5)	92.1 (12.2)	93.7 (12.3)	NS
SBP, mm Hg	128.5 (13.2)	130.1 (14.1)	NS	128.5 (12.7)	126.53 (11.5)	131.1 (14.3)	0.02
DBP, mm Hg	73.1 (7.4)	72.3 (8.7)	NS	71.8 (7.8)	71.8 (7.6)	73.5 (8.6)	NS
Biochemical markers							
NOx, µM	42.62 (23.9)	39.62 (26.74)	NS	41.29 (25.30)	51.73 (32.03)	35.50 (18.35)	0.000
Endothelin-1, pg/ml	1.72 (0.8)	1.73 (0.36)	NS	1.68 (0.63)	1.72 (0.84)	1.66 (0.47)	0.005
TC, mmol/L	4.30 (1.1)	4.23 (1.04)	NS	4.13 (1.08)	4.45 (1.08)	4.24 (1.08)	NS
Triglycerides, mmol/L	1.55 (0.96)	1.57 (0.61)	NS	1.58 (0.90)	1.54 (0.85)	1.50 (0.72)	NS
LDL-cholesterol, mmol/L	2.45 (0.92)	2.46 (0.88)	NS	2.33 (0.90)	2.62 (0.97)	2.43 (0.91)	NS
HDL-cholesterol, mmol/L	1.20 (0.31)	1.06 (0.27)	NS	1.10 (0.31)	1.18 (0.32)	1.12 (0.30)	NS
Glucose, mmol/L	6.12 (1.53)	6.53 (1.91)	0.000	6.32 (1.79)	5.85 (1.35)	6.60 (1.78)	0.000
Insulin, µIU/ml	12.01 (10.7)	14.99 (14.22)	0.000	12.90 (9.82)	10.94 (10.18)	14.55 (12.89)	0.002
HOMA-IR	3.58 (4.21)	4.75 (5.83)	0.002	3.82 (3.66)	2.96 (3.26)	4.64 (5.4)	0.000
CRP, mg/L	5.70 (16.3)	7.98 (12.6)	0.000	9.46 (22.23)	6.80 (21.62)	7.48 (15.72)	NS
Fibrinogen, g/L	4.60 (1.35)	4.88 (1.21)	0.01	5.03 (1.40)	4.59 (1.40)	4.87 (1.25)	0.01
Adiponectin, µg/mL	8.80 (3.61)	8.55 (4.72)	NS	8.86 (5.01)	9.30 (4.55)	9.08 (5.93)	NS
Leptin, ng/mL	44.18 (46.2)	29.04 (38.21)	NS	34.00 (42.50)	39.14 (43.99)	34.78 (41.94)	NS
CDH3, pg/mL	3.50 (2.68)	4.29 (2.96)	0.016	4.02 (2.88)	2.88 (2.72)	4.72 (2.76)	0.000
Statin treatment before blood withdrawal, %	49.3	65.2	0.006	58.2	32.3	72.3	0.000

* 3-year follow-up CV events reported in 80% of the cohort by phone survey included (CV death, ischemic stroke, acute myocardial infarction, unplanned revascularization at least 3 months after discharge from hospitalization, coronary artery bypass grafting, and hospitalization related to cardiovascular events). ** Smoking status: 0, never smoked; 1, smoked in the past; 2, present smoker. WC, waist circumference; LDL, low-density lipoproteins; HDL, high-density lipoproteins; HOMA-IR, homeostatic model assessment insulin resistance; NOx, total concentration of the NO_3_^−^ and NO_2_^−^ ions; CDH3, cadherin-P; SBP, systolic blood pressure; DPB, diastolic blood pressure; BMI, body mass index; TC, total cholesterol; CRP, C-reactive protein; NS, not significant. *** Patients with CV family history: 0—no pathogenic heredity burden; 1—CV diseases were documented in members of immediate family (parents; brothers/sisters).

**Table 2:** Associations between the levels of CDH3 and combined CV outcomes* defined by ROC-analysis and OR evaluation.

	Statistical Method		*p*		
N total = 172; CV outcomes * N = 95; CDH3 (continuous)	AUC (95% CI)	0.58 (0.52–0.64)	0.017	Optimal cut-off for CDH3 = 4.6 pg/mL	Sensitivity 0.55 Specify 0.63
CV outcomes N (50/45); CDH3 (binary) N (53/24); cut-off 4.6 pg/mL	OR (95% CI)	1.81 (1.07–3.72)	0.022		

* 3-year follow-up combined outcomes reported in 80% of the cohort by phone survey included ischemic stroke, acute myocardial infarction, unplanned revascularization at least 3 months after discharge from hospitalization, coronary artery bypass grafting, and hospitalization related to cardiovascular events. CV death was excluded from the outcomes.

**Table 3:** Binomial logistic regression for associations between cardiovascular outcomes * (dependent variable) and serum CDH3 adjusted to routine cardiovascular risk factors in patients after a 3-year follow-up.

Parameters	B	Wald (Chi-Squared)	*p*
Sex	1.251	0.720	0.396
Age	0.430	30.021	0.082
Smoking	−0.038	40.713	0.030
Patients with CV family history **	−0.544	0.781	0.377
Gensini score	0.407	70.346	0.007
Systolic blood pressure, mm Hg	−0.021	40.058	0.044
Diastolic blood pressure, mm Hg	−0.039	50.353	0.021
Total cholesterol, mmol/L	0.078	0.846	0.358
Triglycerides, mmol/L	590.197	0.840	0.359
LDL holesterol, mmol/L	−270.063	0.856	0.355
HDL cholesterol, mmol/L	−590.539	0.764	0.382
Glucose, mmol/L	−560.314	30.088	0.079
C-reactive protein, mg/L	−0.211	0.889	0.346
CDH3, pg/mL	0.013	30.726	0.049

* 3-year follow-up combined outcomes reported in 80% of the cohort by phone survey included ischemic stroke, acute myocardial infarction, unplanned revascularization at least 3 months after discharge from hospitalization, coronary artery bypass grafting, and hospitalization related to cardiovascular events. CV death was excluded from the outcomes. ** Patients with CV family history: 0—no pathogenic heredity burden; 1—CV diseases were documented in members of immediate family (parents; brothers/sisters). LDL, low-density lipoproteins; HDL, high-density lipoproteins; CDH3, cadherin-P.

**Table 4:** Associations of CDH3 on fitting quality assessed by classification tables. The tables were obtained by binomial fitting and calculations of the aggregated dichotomous values, which included routine cardiac risk and CV outcomes * (base model) or base model plus CDH3 at a cut-off of 4.5 pg/mL (base model plus CDH3). Classification Table for Base Model Including Sex, Age at a Median Cut-Off of 65 Years, Patients with CV Family History (Yes/No), Smoking, Systolic Blood Pressure at a Median Cut-Off of 130 mm Hg, Diastolic Blood Pressure at a Median Cut-Off of 70 mm Hg, and Total Cholesterol at a Median Cut-Off of 4.2 mmol/L.

**Observed**	**Predicted**		
	**0**	**1**	**% of Corrected Observations**
0	29	48	37.7%
1	20	75	78.9%
Total %	28.5%	71.5%	60.5%
**Classification Table for Base Model Plus CDH3**			
	**0**	**1**	**% of Corrected Observations**
0	37	36	50.7%
1	21	66	75.9%
Total %	36.2%	63.7%	64.4%

* 3-year follow-up combined outcomes reported in 80% of the cohort by phone survey included ischemic stroke, acute myocardial infarction, unplanned revascularization at least 3 months after discharge from hospitalization, coronary artery bypass grafting, and hospitalization related to cardiovascular events. CV death was excluded from the outcomes.

**Table 5:** Risk reclassification for evaluating prediction models for CV outcomes * and traditional cardiac risk factors (base model) and CV outcomes and CDH3 added to the base model. For details, see Table 4.

Parameters	N	OR (95% CI)	*p*
Routine cardiac risk factors **	29/48 20/75	2.26 (1.15–4.45)	0.017
Routine cardiac risk factors plus CDH3 (cut-off 4.6 pg/mL)	37/36 21/66	3.23 (1.64–6.32)	0.0006

* 3-year follow-up combined outcomes reported in 80% of the cohort by phone survey included ischemic stroke, acute myocardial infarction, unplanned revascularization at least 3 months after discharge from hospitalization, coronary artery bypass grafting, and hospitalization related to cardiovascular events. CV death was excluded from the outcomes. ** Routine cardiac risk factors are listed in Table 4.

**Table 6:** Associations between the CDH3 levels as binary variable (cut-off 4.6 pg/mL) and various parameters in the total cohort defined by ROC analysis.

	AUC (95% CI) for Binary CDH3 (Cut-off 4.6 pg/mL)	Optimal Cut-Off for the Corresponding Parameters According to AUC	*p*
General characteristics			
Sex	-		NS
Age	-		NS
Smoking (0/1/2) **, %			NS
Patients with CV family history.	0.61 (0.52–0.69)		0.016
BMI, kg/m^2^	-		NS
WS, cm	-		NS
SBP, mm Hg	-		NS
DBP, mm Hg	0.58 (0.53–0.63)	71.0	0.006
Biochemical markers			
NOx, µM and endothelin-1 in model	0.64 (0.54–0.74)	33.01	0.005
Endothelin-1, pg/mL	0.66(0.57–0.75)	1.67	0.001
TC, mmol/L	-		
Triglycerides, mmol/L	-		NS
LDL-cholesterol, mmol/L	-		NS
HDL-cholesterol, mmol/L	-		NS
Glucose, mmol/L	-		NS
Insulin, µIU/mL	-		NS
HOMA-IR	-		NS
CRP, mg/L	-		NS
Fibrinogen, g/L	-		NS
Adiponectin, µg/mL	-		NS
Leptin, ng/mL	-		NS
Statins treatment before blood withdrawal, %			NS

For details, see the legend to Table 1. ** Smoking status: 0, never smoked; 1, smoked in the past; 2, present smoker.

**Table 7:** Binomial logistic regression for the associations of NOx and endothelin-1 with CDH3 as binary variable at a cut-off of 0.52 pg/mL.

Parameter	B	S.E.	Wald	df	*p*	Exp (B)
NOx, µM	0.006	0.007	0.855	1	0.355	1.006
Endothelin-1 (pg/mL)	0.854	0.301	8.055	1	0.005	2.349

For details see legend to Table 1.

**Table 8:** Linear regression analysis for systolic and diastolic blood pressure associations with endothelin-1, NOx, and CDH3.

		Unstandardized Coefficients		Standardized Coefficients	*p*
Dependent Variable	Models	B	S.E.	Beta	
Systolic blood pressure, mm Hg	Endothelin-1, pg/mL	−0.687	1.382	−0.041	0.620
	NOx, µM	−0.054	0.027	−0.162	0.045
	CDH3, pg/mL	0.158	0.315	0.041	0.616
Diastolic blood pressure, mm Hg	Endothelin-1, pg/mL	−1.995	0.931	−0.172	0.034
	NOx, µM	−0.016	0.018	−0.069	0.382
	CDH3, pg/mL	0.561	0.212	0.212	0.009

## Data Availability

Data are contained within the article.
